# Intramedullary Thoracic Spinal Cord Abscess Mimicking an Intramedullary Tumor: A Case Report

**DOI:** 10.7759/cureus.43387

**Published:** 2023-08-12

**Authors:** Naeem Ul Haq, Mujtaba Hassan, Zeeshan Ali, Abdullah Abdullah

**Affiliations:** 1 Neurological Surgery, Mardan Medical Complex, Mardan, PAK; 2 Medicine, Khyber Medical University, Peshawar, PAK

**Keywords:** thoracic myelopathy, thoracic spinal cord, neuro infectious diseases, intramedullary abscess, spinal abscess

## Abstract

An intramedullary abscess is an extremely rare form of infection of the spinal cord, with only about 100 cases reported in the literature. It typically presents with back pain, neurological deficits, and, occasionally, fever. The purpose of this article is to report a case of an intramedullary thoracic spinal cord abscess that was initially misdiagnosed as an intramedullary tumor.

A 48-year-old female presented with chronic inter-scapular pain and lower limb weakness. The patient was initially misdiagnosed as a case of intramedullary spinal cord tumor, primarily due to the rarity of intramedullary spinal cord abscess (ISCA) as well as the absence of ring enhancement in the lesion on MRI. However, during a surgical procedure for the excision of the lesion, it was found to be a case of ISCA. The purulent contents of the lesion were evacuated, and the patient was treated with IV antibiotics, leaving the patient with a slight residual lower limb weakness on the follow-up examination.

This case highlights the importance of considering intramedullary abscess as a possible diagnosis in patients with back pain and neurological deficits, even in the absence of ring enhancement on imaging. Prompt surgical intervention should be considered in probable cases of intramedullary spinal cord tumors.

## Introduction

Intramedullary spinal cord abscess (ISCA) is a rare condition that can be easily mistaken for intramedullary tumors. The cervical location is the least commonly affected, except in cases of cryptogenic spread. The accurate diagnosis of ISCA relies heavily on the detailed examination of contrast-enhanced MRI images [1].

ISCA can result in significant neurological damage and even death. The majority of cases arise from a direct source of infection, such as vertebral osteomyelitis or postsurgical infections. However, in cases where no source is found, the infection is known as "cryptogenic" and is associated with risk factors such as immunosuppression, intravenous drug use history, poorly controlled diabetes mellitus, or pulmonary arteriovenous malformations. In adults without these risk factors, cryptogenic ISCA is rare and can be easily misdiagnosed as more common conditions such as spinal intramedullary neoplasia, demyelinating disease, resolving hematoma, or infarction [2].

A spinal cord tumor can be suspected based on contrast-enhanced MRI images, but surgery can reveal a spinal cord abscess. While spinal abscesses are occasionally seen in pediatric patients, intramedullary abscesses are extremely rare [3].

An intramedullary spinal cord abscess is an uncommon infection of the spinal cord resulting in a purulent cystic lesion in the spinal cord substance. The common presentation is fever, pain, neurological deficits, and bladder/bowel dysfunction. The diagnosis is made through MRI, showing a hyperintensity on T2 diffusion-weighted imaging (DWI) and a hypointensity with a peripheral hyperintense rim on gadolinium post-contrast T1 DWI. Prompt diagnosis and management are necessary to avoid or limit permanent damage to the cord. The recommended course of treatment is a combination of surgical intervention for decompression and drainage and culture-specific antimicrobial therapy, though some cases have responded to antimicrobial therapy alone [4]. Here, we present the case of a 48-year-old female patient with ISCA misdiagnosed as an intramedullary tumor due to atypical imaging findings as well as due to her indolent presentation.

## Case presentation

A 48-year-old female presented to us with complaints of progressive bilateral lower limb weakness for the past five months leading up to the presentation and non-radiating midline inter-scapular pain for about three months that was intermittently exacerbated with physical activity. She had no history of any recent infection, immunocompromised state, immunosuppressant drugs, IV drug abuse, previous surgery, or trauma. Her postoperative HIV investigations came back negative. She denied experiencing any bowel or bladder dysfunction. On physical examination, she was found to be afebrile and had paraparesis with motor power of MRC (Medical Research Council's muscle power assessment scale) grade 2/5 in both lower limbs. All her sensory functions, including proprioception, touch, and vibration, were intact.

**Figure 1:**
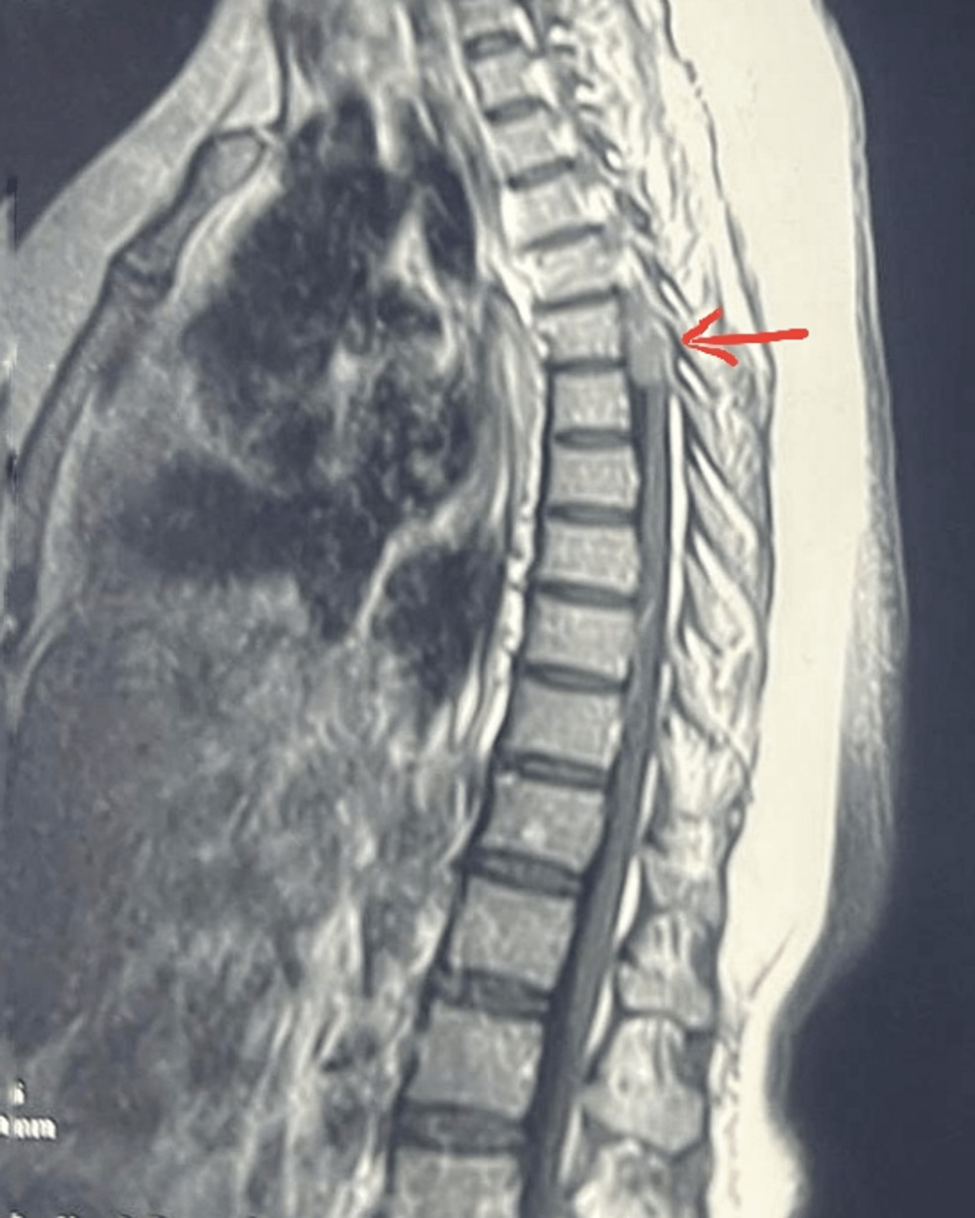
Post-gadolinium contrast T2 diffusion-weighted MRI film showing hyper intense space-occupying lesion at the level of T4-T6 vertebrae

The MRI scan of her spine showed a space-occupying lesion measuring 31 mm at the level of T4-T6 vertebrae, which was hyperintense on T2 diffusion-weighted magnetic resonance imaging (DW-MRI) and hypointense on gadolinium post-contrast T1 DW-MRI, but the latter did not show any peripheral ring enhancement as we would expect for an ISCA (Figure 1). Consequently, she was misdiagnosed as a case of intramedullary tumor with intramedullary astrocytoma and ependymoma on top of our differential diagnoses.

**Figure 2:**
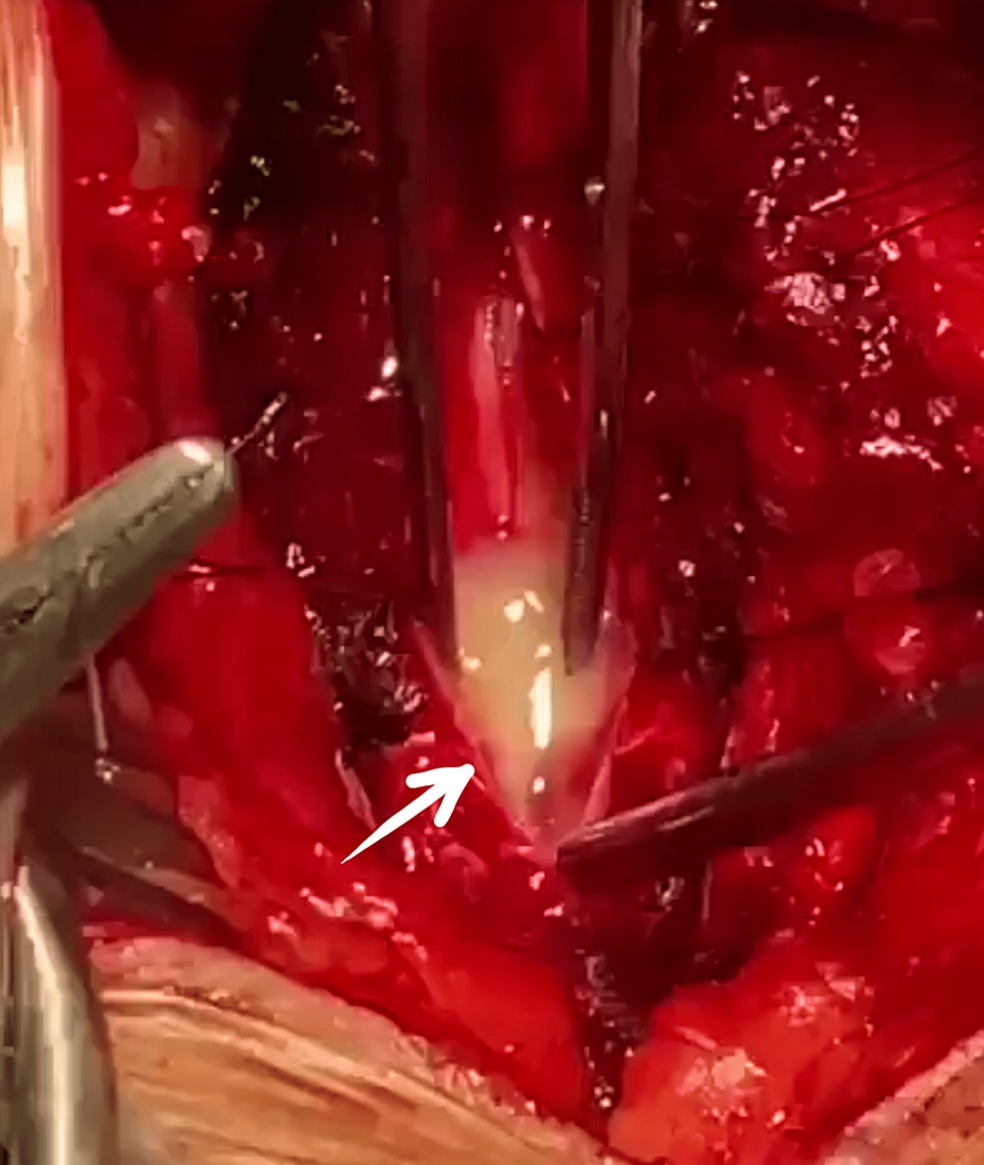
Intraoperative view after myelotomy incision depicting expression of purulent collection

She was started on pre-operative "prophylactic" broad-spectrum antibiotics after consultation with an infectious disease specialist. These included vancomycin and ceftriaxone, and she was scheduled for surgery five days later. During the surgery, laminectomy was performed at T4-T6 levels, and durotomy was done in the standard fashion. This resulted in the bulging of the swollen cord through the durotomy due to the mass effect (Figure 2). A midline myelotomy incision was made, and a whitish purulent fluid leaked out of the lesion, which led to the diagnosis of ISCA. The lesion was evacuated through suction irrigation, and the dura was closed in a watertight fashion. The aspirated sample was sent for bacterial culture, Gram staining, AFB, and antibiotic sensitivity testing, all of which turned out to be negative, probably due to the pre-operative prophylactic antibiotic therapy already in place.

The patient was retained in the inpatient department for four weeks post-operatively to be put on broad-spectrum IV antibiotics (vancomycin and ceftriaxone) for better control of the infection. After that, she was switched to oral antibiotics for the subsequent two weeks. During her course of treatment, she was found to be improving in terms of motor power in her lower limbs. An MRI scan of her dorsal spine showed a significant decrease in the size of the lesion. On her follow-up examination four months post-surgery, she was ambulating with only minor assistance, and her motor power was found to be 4/5 in both lower limbs. She was able to perform activities of daily living with minimal assistance, such as bathing, dressing, and feeding. She scored 2/6 on the Modified Rankin Scale.

## Discussion

ISCA is an extremely rare entity, with only about 100 cases reported [2]. The first description of the condition was given by Hart in 1830 [5]. In a review of 54 ISCA cases described in the literature, significant male predominance has been noted; of the 54 cases, 37 (68.5%) were males, 13 (24.1%) were females, and 4 were not described in the literature [6].

ISCA is characterized by a pus-filled cavity in the spinal cord that more often affects the cervical and lumbar cords and is relatively less common in the thoracic region, as in our case [7]. The relative avascularity and the local compression can together cause significant ischemic damage [5]. The disease may have an acute, subacute, or chronic presentation, with acute cases defined as those presenting with a history of up to one week and subacute ones as those with a history of one to six weeks [8]. In this case, having a history of more than six weeks was classified as chronic ISCA.

Its occurrence is attributed to multiple risk factors, including an immunocompromised state like HIV infection and therapeutic immunosuppression, structural defects like dermal sinuses in the pediatric age group, previous infection, trauma, IV drug abuse, or previous surgery [2]. The source of infection may be hematogenous spread from a distant infection, like a pericolonic abscess, thoracic empyema, or dental infection, or direct seeding from a nearby site of infection, like dermal sinus, spondylodiskitis, or dermoid cyst with dermal sinus tract [4]. In our patient, the CT scans of the thorax, abdomen, and pelvis and transthoracic echocardiogram did not show any signs of neighboring infection, and neither did the patient have any of the said risk factors. The source of infection has not been identified in more than half of the cases of ISCA, including ours, and hence is labeled as cryptogenic [2].

A wide range of microbes have been isolated from intramedullary abscesses, with Mycobacterium tuberculosis being an important one in the developing world [9]. Other causative agents include Streptococci, Staphylococci, and other microbes constituting the skin and oral flora [10].

ISCA is believed to present with a triad of fever, pain, and neurological deficits, though all these findings may not always be observed together in all patients [4]. Other manifestations include bowel and bladder dysfunction (retention or incontinence), night sweats, chills, recurrent meningitis, and a dorsal midline skin lesion if associated with a dermal sinus, as usually seen in pediatric patients. Our patient lacked most of these manifestations and was only experiencing back pain and neurological deficits in the form of paraparesis.

The diagnosis is made through history, clinical features, and magnetic resonance imaging. A ring-enhancing lesion on T1 DW-MRI is thought to be strongly suggestive of ISCA, but the absence of ring enhancement does not rule out ISCA, as we discovered in our case. The presence of the drop sign or precipitation sign (a new radiological sign for the diagnosis of spinal abscess, especially tubercular spinal abscess, characterized by an accumulation of pus in the conus medullaris) has also been reported, which, if present, may give a better clue to ISCA. A CSF examination may reveal elevated proteins, normal to low glucose levels, and leukocytosis [9].

Concerning treatment, ISCA is best treated with a combination of surgery and antimicrobial therapy, though antibiotics alone may be effective in some cases [4]. Antibiotic therapy should be started preoperatively in diagnosed cases of ISCA; surgical drainage and decompression should be performed; and the antibiotic regimen should be narrowed down according to sensitivity reports. The recommended duration of medical treatment is the same as for a brain abscess, i.e., six weeks [9]. The mortality rate has fallen to 8% in the antibiotic era as opposed to 90% in the pre-antibiotic era [10]. Though the mortality rate is higher for acute ISCA, if treated early, residual neurological deficits are relatively less common in acute ISCA as compared to subacute and chronic cases [4].

## Conclusions

The authors highlight the significance of considering ISCA as a potential diagnosis, despite its rarity, when faced with cases that resemble intramedullary tumors, particularly in instances where no ring enhancement is observed on T1 DW-MRI. Given the challenging nature of distinguishing between these conditions, the inclusion of ISCA in the differential diagnosis becomes crucial to ensuring appropriate and timely management. Failure to recognize and address ISCA promptly may lead to irreversible cord damage, which could result from an underlying, yet undetected, abscess. Therefore, the authors stress the importance of maintaining a high index of suspicion and employing thorough diagnostic evaluation to avoid overlooking ISCA and facilitate immediate surgical intervention when intramedullary tumors are suspected, thus mitigating the risk of potential long-term consequences.

The authors strongly advocate for immediate surgical intervention in cases where intramedullary tumors are diagnosed to prevent the possibility of inadvertent harm to the spinal cord caused by a potentially concealed ISCA. Given the delicate and intricate nature of spinal cord pathologies, the urgency of accurate diagnosis and prompt treatment cannot be understated. The presentation of an ISCA without ring enhancement on T1 DW-MRI highlights the diagnostic challenges involved, making surgical exploration and intervention imperative to safeguard the patient's neurological function. By emphasizing the necessity for early surgical management in such cases, the authors aim to ensure that appropriate measures are taken swiftly to address the underlying pathology, mitigating the risk of irreversible cord damage and improving the overall prognosis for the patient.
